# Derotational distal femoral osteotomy for patients with recurrent patellar instability and increased femoral antetorsion improves knee function and adequately treats both torsional and valgus malalignment

**DOI:** 10.1007/s00167-022-07150-9

**Published:** 2022-09-16

**Authors:** Maximilian Hinz, Matthias Cotic, Theresa Diermeier, Florian B. Imhoff, Georg C. Feuerriegel, Klaus Woertler, Alexander Themessl, Andreas B. Imhoff, Andrea Achtnich

**Affiliations:** 1 Department of Sports Orthopaedics, Technical University of Munich, Ismaninger Street 22, 81675 Munich, Germany; 2 Unfallkrankenhaus Berlin, Berlin, Germany; 3 Rennbahnklinik, Muttenz, Switzerland; 4 Musculoskeletal Radiology Section, Technical University of Munich, Munich, Germany

**Keywords:** Patellofemoral instability, Derotational distal femoral osteotomy, Femoral antetorsion, Torsional deformity, Patellar dislocation, Patient-reported outcome measures, Functional outcome

## Abstract

**Purpose:**

The aim of the study was to evaluate the functional and radiological outcome following derotational distal femoral osteotomy (D-DFO) in patients with high-grade patellofemoral instability (PFI) and an associated increased femoral antetorsion (FA). It was hypothesized that D-DFO would lead to a good functional and radiological outcome, and that both torsional and coronal malalignment could be normalized.

**Methods:**

Patients that underwent D-DFO between 06/2011 and 12/2018 for high-grade PFI with an increased FA (> 20°) were included. Patient-reported outcome measures (Visual Analog Scale [VAS] for pain, Kujala score, Lysholm score, International Knee Documentation Committee subjective knee form [IKDC], and Tegner Activity Scale [TAS]) were evaluated pre- and minimum 24 months postoperatively. Magnetic resonance imaging of the lower extremity and weight-bearing whole-leg anteroposterior radiographs were conducted pre- and postoperatively. The change in FA, coronal limb alignment, and PROMs were tested for statistical significance.

**Results:**

In total, 27 patients (30 knees) were included. The D-DFO aimed to only correct FA (Group 1) or to additionally perform a varization (Group 2) in 14 cases each. In the remaining two cases, double-level osteotomies were performed to correct additional tibial deformities. In 25 cases (83.3%), concomitant procedures also addressing patellofemoral instability were performed. At follow-up (38.0 months [25–75% interquartile range 31.8–52.5 months]), a significant reduction in pain (VAS for pain: 2.0 [1.0–5.0] vs. 0 [0–1.0], *p* < 0.05), significant improvement in knee function (Kujala Score: 55.6 ± SD 13.6 vs. 80.3 ± 16.7, *p* < 0.05; Lysholm Score: 58.6 ± 17.4 vs. 79.5 ± 16.6, *p* < 0.05; IKDC: 54.6 ± 18.7 vs. 74.1 ± 15.0, *p* < 0.05), and an increase in sporting activity (TAS: 3.0 [3.0–4.0] vs. 4.0 [3.0–5.0], *p* = n.s.) were reported. Femoral antetorsion was significantly reduced (28.2 ± 6.4° vs. 13.6 ± 5.2°, *p* < 0.05). A significant varization was observed in Group 2 (2.4 ± 1.2° valgus vs. 0.3 ± 2.4° valgus; *p* < 0.05). In one case, patellar redislocation occurred 70 months postoperatively.

**Conclusion:**

In patients with PFI and an associated increased FA, D-DFO achieved a significant reduction in pain, an improvement of subjective knee function, as well as an adequate correction of torsional and coronal alignment.

**Level of evidence:**

Retrospective case series, Level IV.

## Introduction

Patellofemoral instability (PFI) is often a multifactorial problem that can result from a combination of coronal limb malalignment, torsional deformities of the femur and tibia, trochlear dysplasia, patella alta, or disrupted and weakened medial soft tissue, including the medial patellofemoral ligament (MPFL) and the vastus medialis obliquus [2, 18].

Reconstruction of the MPFL is considered the gold standard in the treatment of recurrent PFI, achieving excellent results with a reported risk for recurrent instability of less than 2% [16]. In complex cases of PFI, however, failure to address the aforementioned risk factors may negatively affect the outcome, and thus, a different treatment strategy may be necessary [5, 12, 13]. Additionally, it has been shown that an increased FA leads to a lateral patellar shift during motion, indicating that an isolated MPFL reconstruction may not be feasible in patients with PFI and FA ≥ 20° [9, 10].

Derotational distal femoral osteotomy (D-DFO) has been proposed as a treatment option for recurrent PFI with an associated increased FA with favorable outcomes observed at short-term follow-up [20]. To our knowledge, while previous clinical studies have reported the postoperative change in FA following D-DFO, they did not assess the change in coronal limb alignment or did so within a limited scope [3, 20]. Examining this change is important to accurately appraise D-DFO as a treatment option, since a postoperative valgus deviation may increase the risk of instability recurrence.

The purpose of the present study was to evaluate the clinical, functional, and radiological outcome following D-DFO in patients with recurrent PFI and increased FA (> 20°) after a minimum follow-up of 24 months. It was hypothesized that: 1) D-DFO would lead to a significant improvement in subjective knee function with a low rate of patellar redislocation and a significant reduction in FA, 2) in cases of preoperative straight coronal limb alignment, no significant change in coronal alignment—especially no severe, unintended valgus deformity—would occur postoperatively, and finally, 3) in cases of preoperative valgus limb alignment, an additional varization would be achievable through the surgical procedure.

## Materials and methods

The present study was approved by the ethics committee of the Technical University of Munich (reference: 110/18 S) and conducted according to the Declaration of Helsinki. All patients gave their written and informed consent.

Patients that underwent D-DFO between 06/2011 and 12/2018 at our institution for the treatment of recurrent PFI with an associated increased FA at a minimum of 24 months postoperatively were included in the clinical, functional and radiological assessment. Only patients with no previous alignment-correcting surgeries and a minimum age of 18 years at follow-up were included.

Patient-reported outcome measures (PROMs; Visual Analog Scale for pain [VAS], Kujala Score, Lysholm Score, International Knee Documentation Committee subjective knee form [IKDC], and Tegner Activity Scale [TAS]) were conducted pre- and minimum 2 years postoperatively. Additionally, range of motion (ROM) was evaluated preoperatively and at follow-up. Demographic factors and patient records with a special focus on postoperative injury recurrence and complications were analyzed. Pre- and postoperative imaging included lower extremity and torsional magnetic resonance imaging (MRI) as well as full weight-bearing whole-leg radiographs.

### Radiological parameter measurements and surgical planning

Postoperatively, all patients underwent 3 T MRI of the lower limb including a 3D T1-weighted turbo spin echo sequence which was reformatted in sagittal, axial, and coronal orientation. Femoral antetorsion was assessed using a method previously described by Schneider et al. [15]. In detail, FA was defined as the angle between a line connecting the center of the femoral head, the center of the distal femoral neck (femoral neck axis), and the distal femur (dorsal margin of the femoral condyles). External tibial torsion was defined as an angle between a tangent drawn along the dorsal margin of the proximal tibia and, distally, a line connecting the center of the tibial pilon with the center of a line across the fibular incision of the distal tibia.

For the analysis of coronal limb alignment, the femorotibial angle, mechanical lateral distal femoral angle (mLDFA), and mechanical medial proximal tibial angle (mMPTA) were analyzed on weight-bearing whole-leg anteroposterior radiographs using the medical software mediCAD® (accuracy: 0.01°; mediCAD Hectec GmbH, Altdorf, Germany) according to the method proposed by Strecker [17].

A D-DFO was indicated in patients suffering from recurrent patellar dislocation with an associated increased FA > 20° and closed physes (Group 1). In patients with an additional valgus deformity, coronal limb malalignment correction was performed simultaneously (Group 2). The cut-off values for whether a valgus correction was performed varied between surgeons as current literature does not provide a real cut-off value for a relevant valgus deformity.

Furthermore, additional procedures, including MPFL reconstruction, tibial tuberosity transfer, and trochleoplasty, were performed based on an individual risk factor analysis.

All torsion measurements were conducted by 2 independent examiners (M.H. and G.C.F.) according to the method by Schneider et al. [15] to evaluate the intraclass correlation coefficient and, subsequently, calculate the interrater reliability.

### Surgical technique

Diagnostic arthroscopy was performed first to evaluate the cartilage, patellar tracking, and the integrity of the medial retinaculum. In cases of a high lateralization tendency following D-DFO, an additional reconstruction of the MPFL using the ipsilateral gracilis tendon was performed.

A biplanar supracondylar torsional osteotomy of the femur was performed via a standardized lateral subvastus approach. A detailed description of the authors’ preferred operative technique was previously described [8]. The degree of torsional correction was controlled via two bicortically placed Kirschner wires or Steinman nails proximal and distal to the osteotomy from an axial view. For fixation of the osteotomy, an internal plate fixator system with locking screws was used (Tomofix distal femoral plate, dePuy Synthes, Umkirch, Germany). The aim of the coronal correction was to achieve neutral alignment.

### Postoperative rehabilitation

Postoperatively, partial weight-bearing (20 kg) for 6 weeks was allowed. The degree of ROM restriction was dependent on the presence of any additional surgical procedures. If an additional MPFL reconstruction was performed, ROM was limited to 90° of flexion for the first 6 weeks. Following check-up at 6 weeks postoperatively, full weight-bearing was encouraged. Physical therapy started on the first postoperative day with passive flexion and continued 2–3 times per week thereafter.

### Statistical analysis

Data were analyzed using SPSS 26.0 (IBM-SPSS, New York, USA). Categorical variables are presented in counts and percentages. Normal distribution of the collected continuous variables was assessed by the Shapiro–Wilk test and graphically confirmed. Normally distributed continuous variables are shown as mean ± standard deviation. Non-normally distributed continuous variables are shown as median (25–75% interquartile range). For group comparisons of continuous variables, the Wilcoxon test or *t* test was applied. Spearman's rank-order correlation was used to assess the correlation between the patient’s age and the postoperative outcome. Statistical significance was set at a *p* value of < 0.05.

The intraclass correlation coefficient was calculated to assess the interrater reliability of FA measurements. For all torsion measurements, interrater reliability was scored as “substantial” to “almost perfect” (0.777–0.902).

To assess the statistical power of this study, a post hoc power analysis was performed for the pre- to postoperative change in FA and Kujala score using two-tailed *t* tests. It was shown that the included sample size could achieve an adequate power of > 0.999 with an alpha of 0.05 (G*Power 3.1.9.6, Düsseldorf, Germany) [4].

## Results

Follow-up examinations, including postoperative lower extremity MRI, were conducted in 27 patients (30 knees). Of those 27 patients, 13 (43.3%) underwent previous surgeries before undergoing D-DFO at the authors’ institution. Follow-up examinations were conducted 38.0 months (31.8–52.5 months) postoperatively. Detailed patient demographics are shown in Table 1.

**Table 1:** Patient demographics

	Mean ± SD or No*.* (%)
No. of knees/patients	30/27
No. of patients with bilateral D-DFO	3 (11.1%)
Sex (female/male)	23/4 (85.2% female)
BMI (kg/m^2^)	24.3 ± 4.7
Age at time of surgery (years)	23.5^a^ (19.8–29.0)
No. of knees with previous surgeries for PFI and amount of previous knee surgeries	13 knees (43.3%) 2.0^a^ (1.0–2.5)
Follow-up (months)	38.0^a^ (31.8–52.5)

Normally distributed continuous variables are shown as mean ± standard deviation. Non-normally distributed continuous variables are shown as median (25–75% interquartile range)

*D-DFO* Derotational distal femoral osteotomy

^a^Values are median

### Surgical procedures

The D-DFO aimed to only correct FA (Group 1) or to perform an additional varization (Group 2) in 14 cases (46.7%) each. In two cases (6.7%), a two-stage double-level osteotomy was performed. In these cases, high tibial osteotomies (1 × derotation to correct tibial malrotation, 1 × medial closing-wedge to correct a pathological mMPTA in a varus knee) were performed following an FA-correcting D-DFO. In 25 cases (83.3%), concomitant procedures were performed either at the time of the initial procedure (17 cases; 56.7%) or in two stages (8 cases; 26.7%), usually at the time of hardware removal. An overview of all concomitant procedures performed is shown in Table 2. Up until the follow-up examination, implant removals were performed in 27 knees (90.0%). One patellar redislocation occurred 5 years and 10 months postoperatively. Reosteosyntheses were performed in three cases (10%) due to implant failure.

**Table 2:** Concomitant procedures (calculation is based on number of knees [*n* = 30])

	No. (%)
MPFL-Reconstruction	21 (70.0%)
Lateral patellar retinacular lengthening	3 (10.0%)
Trochleoplasty	3 (10.0%)
Double-level osteotomy	2 (6.7%)
Derotational high tibial osteotomy	1 (3.3%)
Medial closing-wedge high tibial osteotomy	1 (3.3%)
Patellar lateral facetectomy	2 (6.7%)
Medialization/distalization of the tibial turbercle	1 (3.3%)
Vastus medialis oblique transfer	1 (3.3%)

*MPFL* Medial patellofemoral ligament

### Patient-reported outcome measures and clinical outcome

Knee function as well as pain levels improved significantly from pre- to postoperative (*p* < 0.05). Sporting activity, assessed via TAS, increased, however not statistically significantly (*p* = n.s.; Table 3). No differences were found when pre- vs. postoperative knee ROM or postoperative vs. contralateral knee ROM were compared (*p* = n.s.; Table 4). No significant correlation was found between the patients’ age and PROMs (*p* = n.s.).

**Table 3:** Patient-reported outcome measures (PROMs)

PROMs	Preoperative	At follow-up	*p* value
VAS	2.0^a^ (1.0–5.0)	0^a^ (0–1.0)	** < 0.05**
TAS	3.0^a^ (3.0–4.0)	4.0^a^ (3.0–5.0)	n.s
Lysholm Score	58.6 ± 17.4	79.5 ± 16.6	** < 0.05**
IKDC	54.6 ± 18.7	74.1 ± 15.0	** < 0.05**
Kujala Score	55.6 ± 13.6	80.3 ± 16.7	** < 0.05**

Normally distributed continuous variables are shown as mean ± standard deviation. Non-normally distributed continuous variables are shown as median (25–75% interquartile range)

Bolded *p* values indicate statistical significance

*IKDC* International Knee Documentation Committee subjective knee form, *PROMs* Patient-reported outcome measures, *TAS* Tegner Activity Scale, *VAS* Visual Analog Scale for pain

^a^Values are median

**Table 4:** Physical examination results

	Preoperative	At follow-up	*p* value
Passive ROM knee flexion - operated leg (°)	140.0^a^ (130.0–140.0)	140.0^a^ (130.0–147.0)	n.s
Passive ROM knee extension - operated leg (°)	0.0^a^ (0.0–5.0)	0.0^a^ (0.0–5.0)	n.s

	Operated leg	Contralateral leg	*p* value
Passive ROM knee flexion at follow-up (°)	140.0^a^ (130.0–147.0)	147.5^a^ (130.0–150.0)	n.s
Passive ROM knee extension at follow-up (°)	0.0^a^ (0.0–5.0)	0.0^a^ (0.0–5.0)	n.s

Normally distributed continuous variables are shown as mean ± standard deviation. Non-normally distributed continuous variables are shown as median (25–75% interquartile range)

*ROM* Range of motion

^a^Values are median

### Radiological outcome

**Fig. 1:**
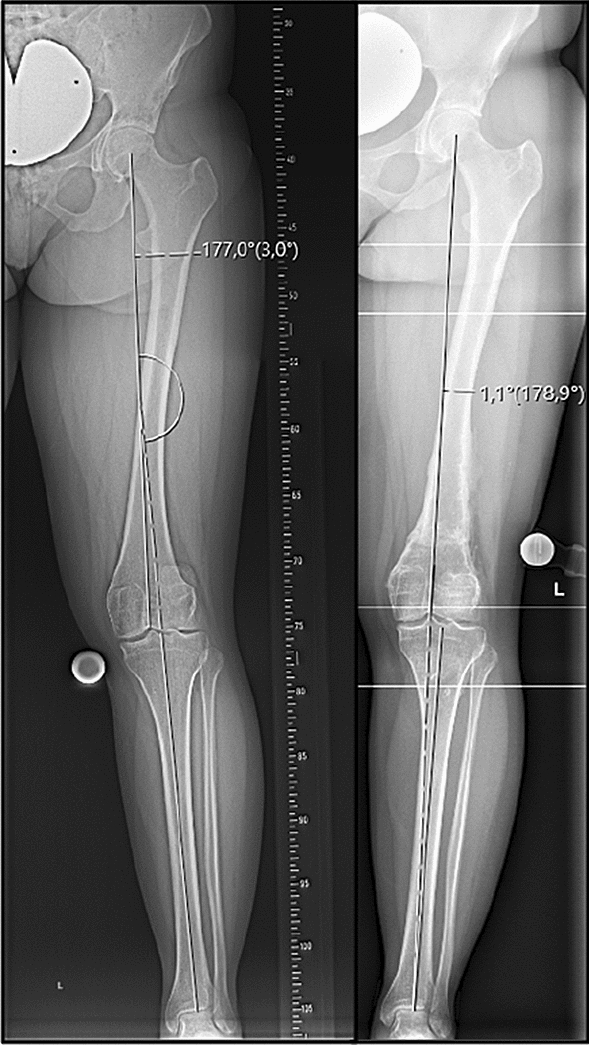
Weight-bearing whole-leg anteroposterior radiographs taken pre- (left) and postoperatively (right) following a derotational distal femoral and valgus-correcting osteotomy showing a sufficient varization

**Fig. 2:**
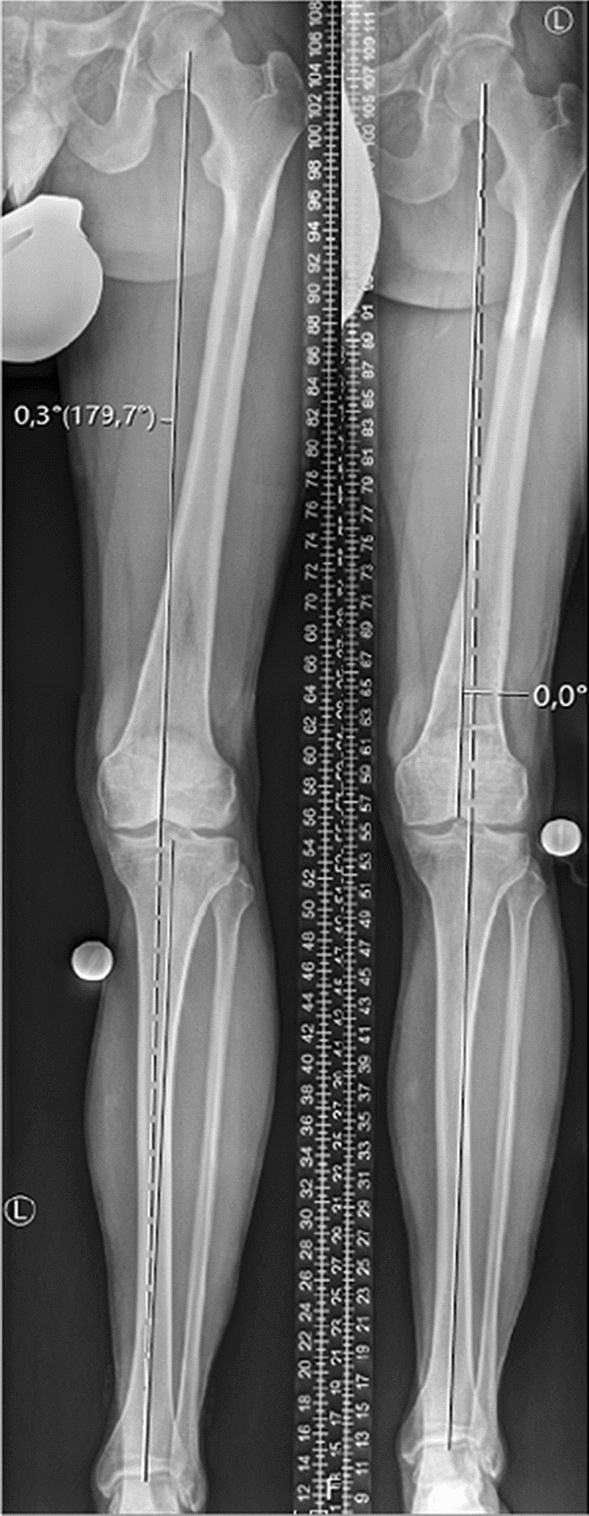
Weight-bearing whole-leg anteroposterior radiographs taken pre- (left) and postoperatively (right) following a derotational distal femoral osteotomy showing no relevant change in coronal limb alignment

Femoral antetorsion was significantly reduced from pre- to postoperatively (28.2 ± 6.4° vs. 13.6 ± 5.2°, *p* < 0.05). A detailed analysis of the individual groups is shown in Table 5. Notably, coronal limb alignment and mLDFA changed significantly in Group 2 (Fig. 1), but did not in Group 1 (Fig. 2).

**Table 5:** Radiological assessment of the coronal limb alignment for both isolated derotational (Group 1) and combined derotational and varization (Group 2) distal femoral osteotomy

	Preoperative	At follow-up	*p* value
Isolated derotational distal femoral osteotomy (Group 1)			
Coronal limb alignment^a^ (°)	− 0.9 ± 2.2	− 1.9 ± 2.0	n.s
Mechanical lateral distal femoral angle (°)	88.7 ± 2.9	89.5 ± 2.9	n.s
Mechanical medial proximal tibia angle (°)	87.8 ± 2.5	87.6 ± 1.8	n.s
Combined derotational and varization distal femoral osteotomy (Group 2)			
Coronal limb alignment^a^ (°)	2.4 ± 1.2	0.3 ± 2.4	** < .05**
Mechanical lateral distal femoral angle (°)	87.3 ± 2.5	89.0 ± 3.5	** < .05**
Mechanical medial proximal tibial angle (°)	88.5 ± 1.4	88.0 ± 1.5	n.s

Normally distributed continuous variables are shown as mean ± standard deviation. Non-normally distributed continuous variables are shown as median (25–75% interquartile range)

Bolded *p* values indicate statistical significance

^a^Positive values indicate valgus alignment; negative values indicate varus alignment

## Discussion

The most important finding of this study was that D-DFO leads to a good clinical, functional, and adequate radiological outcome with a low rate of patellar redislocation in patients with high-grade PFI and increased FA at short- to mid-term follow-up. In cases with straight coronal leg alignment, no significant changes in mLDFA or coronal limb alignment were observed, whereas in patients with a valgus deformity, a significant varization and increase of the mLDFA were observed. In 83.3%, additional surgical procedures were necessary to restore patellar stability.

In the current study, a significant reduction in knee pain, an improvement in knee function and a slight increase in sporting activity level were observed. This aligns with the findings reported in previous studies [20]. Contrary to previous findings, however, a correlation between the patients’ age and postoperative PROMs was not apparent in the current study [19]. The threshold for FA for which a D-DFO was indicated, however, varied between studies, usually ranging from 20 to 30°. This discrepancy might be even more emphasized due to different measurement methods used between studies to determine FA [14]. Biomechanically, FA > 20° has been identified as a significant risk factor for PFI, and in cases of PFI with an associated FA > 20°, an isolated MPFL reconstruction may be insufficient [9, 10]. Clinically however, mixed results have been reported. One study by Blanke et al. [1] showed that an isolated MPFL reconstruction results in favorable clinical outcomes and achieves patellar stability irrespective of FA (< 20° vs. > 20°) [1]. A study by Zhang et al. [21], on the other hand, analyzed the outcome of patients with recurrent PFI and an increased FA (> 30°) who underwent MPFL reconstruction either with or without D-DFO (and with or without tibial tubercle transfer). They reported that a combination of D-DFO and MPFL reconstruction (with or without tibial tubercle transfer) led to a more favorable outcome. Similarly, an increased FA (≥ 30°) has been shown to negatively affect the outcome in patients who underwent either an isolated MPFL reconstruction or MPFL reconstruction and antermedialization of the tibial tubercle [7, 22].

Despite the substantial effort that has been put into reporting on the clinical outcome and change in FA following D-DFO in patients with PFI, there still exists a paucity of data regarding the effect a D-DFO has on coronal limb alignment. A previous study by Nelitz et al. [11] showed that performing a D-DFO may lead to an unintended valgus alignment deviation which should be avoided as a valgus deviation negatively impacts patellar stability [12]. Therefore, care must be taken to avoid an increase in valgus alignment when performing a D-DFO. In the present study, 14 patients underwent a D-DFO that only intended to address the increased FA. At follow-up, no significant changes in coronal limb alignment or mLDFA were observed. These findings affirm those of a recent biomechanical study which showed that no relevant coronal limb alignment deviation may be expected following D-DFO [6]. Additionally, in the present study, 14 patients underwent an additional varization in combination with the D-DFO, which lead to a significant varization as intended. In a cohort of patients that suffered from PFI with an associated increased FA (> 25°) as well as a valgus deformity (≥ 5°), performing a MPFL reconstruction and D-DFO led to a satisfactory clinical outcome and adequately addressed both FA and coronal limb deformity [3]. Therefore, both an isolated D-DFO and a combination of derotation and varization distal femoral osteotomy may be feasible in the clinical setting.

There are some limitations of this study. First, the coexistence of other risk factors for PFI required combined surgical procedures to complement the osteotomy in some cases. Therefore, the results of the D-DFO might be influenced by the additional procedures performed. Nonetheless, this occurrence is representative of the patient cohort. Other limitations include the retrospective nature of the present study and the absence of a control group, which is due in part by the unavailability of alternative treatment options. Furthermore, the median follow-up of 38.0 months may not have been sufficient to evaluate the long-term clinical success rate. Nevertheless, the radiological outcome was favorable, and FA and coronal limb alignment were adequately addressed.

## Conclusion

Patients with high-grade PFI and an associated increased FA can be successfully treated with D-DFO. A significant reduction in pain, improvement of subjective knee function, and an adequate correction of torsional and coronal alignment are achieved at short- to mid-term follow-up.
